# Deciphering the Emergence of Biofilm-Independent Colistin Persistence and Resistance in *A. baumannii*: Toxin–Antitoxin Omics and Novel T/A mRNA-asRNA Balance Regulatory Models

**DOI:** 10.3390/antibiotics15040337

**Published:** 2026-03-26

**Authors:** Eleonora Chines, Ludovica Boscarelli, Gaia Vertillo Aluisio, Maria Santagati, Maria Lina Mezzatesta, Viviana Cafiso

**Affiliations:** 1 Department of Biomedical and Biotechnological Sciences, University of Catania, 95123 Catania, Italy; eleonora.chines01@universitadipavia.it (E.C.); ludovica.boscarelli@studium.unict.it (L.B.); gaia.vertilloaluisio@unict.it (G.V.A.); m.santagati@unict.it (M.S.); mezzate@unict.it (M.L.M.); 2 PhD National Program in One Health Approaches to Infectious Diseases and Life Science Research, Department of Public Health, Experimental, and Forensic Medicine, University of Pavia, 27100 Pavia, Italy

**Keywords:** persistence, dormancy, CRAB, T/A systems, genomics, transcriptomic profiling, col resistance

## Abstract

**Background**: Persistence represents a critical evolutionary reservoir for the development of antimicrobial resistance in *Acinetobacter baumannii* (*Ab*). Understanding the basal mechanisms that enable this survival strategy is crucial for elucidating how high-risk clones evolve resistance during therapy. **Methods**: High-dose colistin time-kill assays were performed in ten ST2 clinical colistin-susceptible (COL-S) Carbapenem-Resistant *Ab* (CRAB) developing in vivo stable and full-colistin resistance to detect persisters. Genomics and basal transcriptomics of chromosomal/plasmid toxin–antitoxin systems (T/As) were performed, as duplicates for each sample, in two ST2 COL-S CRAB to investigate the genomics and basal T/A transcriptomic backgrounds. **Results**: Phenotypically, all strains showed a persistent subpopulation (~1% survival at 8 h) under 5× COL MIC exposure. Genomics identified 10 type-II and one type-IV T/A systems. Basal transcriptomics revealed active expression patterns mainly of GNAT superfamily T/A systems, with consistently low toxin mRNA levels associated with toxin- or antitoxin-directed asRNAs in chromosomal modules. This architecture defined new dual-combined regulatory models in which asRNAs acted as primary T/A mRNA balance modulators, putatively impacting on the T/A mRNA ratio. Conversely, the plasmid-encoded BrnT/A module showed a highly balanced expression. **Conclusions**: Our findings revealed, for the first time, the role of the type-II GNAT T/A superfamily as putative molecular switchers via a fine-tuning transcript balance regulation, impacting the transition from a metabolically active cell state to a dormant one in developing colistin persistence and in vivo resistance CRAB.

## 1. Introduction

*Acinetobacter baumannii* (*Ab*), and mainly Carbapenem-Resistant *Ab* (CRAB), has emerged as one of the most critical nosocomial pathogens globally, currently ranked by the World Health Organization as a critical priority for the research and development of new antimicrobials [1,2]. This therapeutic crisis is a direct consequence of CRAB resistance to carbapenems, the first-line therapy used to treat severe *Ab* infections. Among the last-resort options, colistin is the cornerstone of salvage therapy for CRAB infections, despite significant concerns regarding its nephrotoxicity and neurotoxicity [3].

However, the efficacy of colistin is now alarmingly threatened due to the rapid evolution of colistin resistance during therapy. Indeed, a study previously published by our research group characterized the in vivo emergence of stable, high-level colistin resistance in extensively drug-resistant (XDR) *Ab* clinical isolates [4].

Subsequently, we further elucidated the diverse phenotypic onset strategies and the genomic mosaicism that drives the stability and evolution of colistin resistance within these clinical lineages [5].

This phenomenon of in vivo evolution, in which a susceptible population survives under high antibiotic pressure long enough to acquire stable resistance, points to insidious survival strategies distinct from innate resistance: bacterial tolerance and persistence [6,7].

Tolerance refers to the ability of an entire bacterial population to survive lethal concentrations of antimicrobials for extended periods without changes in their Minimum Inhibitory Concentration (MIC). While tolerance is a population-wide survival strategy, persistence is restricted to a subpopulation characterized by a biphasic killing curve in which the majority of the population rapidly dies, while a resilient fraction persists [6,8]. In contrast to resistance, tolerance and persistence are non-heritable phenotypic traits and not genetically acquired [7].

Persister cells are a small, dormant, and isogenic fraction (typically 10^−2^ to 10^−6^) of the total bacterial population, capable of resuscitation once the antibiotic pressure is removed [8,9].

This metabolic resilience is a major cause of therapeutic failure and chronic, relapsing infections [10]. The molecular switch that actively governs this entry into a dormant, tolerant state is widely attributed to toxin/antitoxin systems (T/As) [11], ubiquitous chromosomic or plasmid genetic loci typically organized as bicistronic operons, composed of a stable toxin targeting essential cellular functions and an unstable antitoxin that counteracts it [12]. In stress-free growth conditions, the antitoxin counteracts the cognate toxin, thereby inhibiting its toxicity. On the contrary, under stress conditions, such as antimicrobial exposure, the antitoxin is either downregulated or degraded by specific ATP-dependent cellular proteases, including Lon and the Clp complex composed of ClpP, ClpX, and ClpS [13,14,15]. The proteolytic activation leads the toxin to exert its inhibitory activity, triggering persisters [13].

While toxins are always stable proteins, T/As are currently classified into eight types based on their antitoxin nature and modality of action. Among these, in types I, III, and VIII, the antitoxin is an RNA that inhibits toxin mRNA translation or sequestrates the protein. Additionally, types II, IV, V, VI, and VII have protein antitoxin and diverse mechanisms of action, ranging from the direct neutralization of the toxin protein through binding (in Type II), to the protection of its cellular targets (Type IV) [12,13,16,17]. However, the precise molecular mechanisms underlying their contributions to persistence in *Ab* are not well characterized.

Despite extensive research on genetic resistance, several knowledge gaps persist regarding the adaptive strategies of XDR *A. baumannii* under antimicrobial pressure. First, although the emergence of stable resistance is well documented, the precise contribution of non-heritable phenotypes, such as bacterial persistence, to the clinical failure of colistin therapies is not well understood. Moreover, although biofilms are known to harbor persister cells, there is a lack of evidence concerning biofilm-independent persistence mechanisms in the globally dominant ST2 lineage.

Our investigation aimed to explore the colistin persistence in ten ST2 clinical XDR COL-S CRAB isolates developing resistance during colistin therapy, alongside the ST2 COL-S *Ab* ACICU reference strain. Moreover, we aimed to address the genomic and basal transcriptomic backgrounds in two selected clinical ST2 XDR COL-S CRAB developing colistin persistence and in vivo resistance under colistin therapy. Specifically, we focused on the genomic architecture and basal transcriptomic profile of T/A systems.

## 2. Results

### 2.1. Antimicrobial Resistance

Ten ST2 clinical COL-S CRAB (1S–10S) isolates were phenotypically characterized and classified as XDR according to Magiorakos et al., 2012 [18]. In broth microdilution MICs, all ST2 CRAB showed resistance to all antimicrobials tested except for tigecycline (TGC) (MIC of 0.5–2 mg/L) and COL (MIC of 2 mg/L). ST2 COL-S *Ab* ACICU was susceptible to COL (MIC of 2 mg/L), MIC for TGC was 4 mg/L, and was resistant to imipenem (IMP), meropenem (MEM), ciprofloxacin (CIP), amikacin (AK), ampicillin-sulbactam (SAM), gentamicin (GEN), and trimethoprim-sulfamethoxazole (SXT).

### 2.2. Colistin Persister and Biofilm Production Assays

In all biological triplicates, clinical ST2 COL-S CRAB 1S–10S and ST2 COL-S *Ab* ACICU showed the presence of a persistent subpopulation induced under the pressure of 5× COL MIC (64 mg/L). This ability was not related to biofilm production ability, as all CRAB were non-biofilm producers (OD_655_ < 0.04).

**Figure 1:**
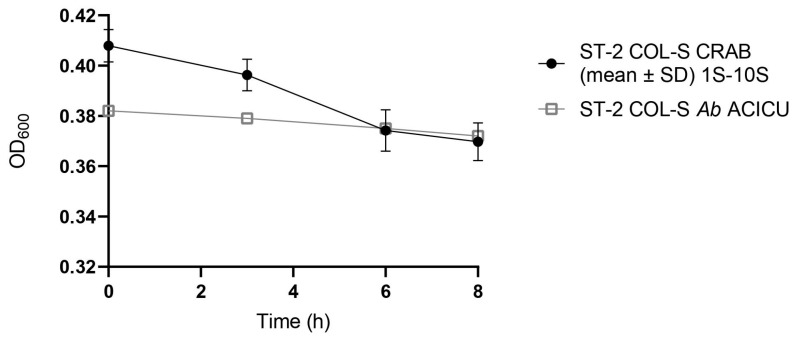
Persister assay OD_600_ curves of ST2 CRAB clinical strains during 8 h of growth under COL selective pressure. **Legend:** Data for ST2 COL-S CRAB are represented by the mean ± SD of the ten clinical isolates (1S–10S; n = 10).

Turbidity measurement (OD_600_) of the colistin persister curves of ST2 COL-S CRAB 1S–10S and ST2 COL-S *Ab* ACICU displayed a constant OD_600_ value ranging from 0.4 to 0.35 during all the T_0_–T_8_ persister assay, as shown in Figure 1. As expected, the OD_600_ values remained relatively stable during assays, as optical density reflects total cell biomass (including both viable and non-viable cells) rather than active bacterial viability.

After COL exposure, strains were plated on Mueller-Hinton Agar (MHA) antimicrobial-free medium at T_3_, T_6_, and T_8_, and a statistically significant reduction in the survivor population was observed in the viability count.

**Figure 2:**
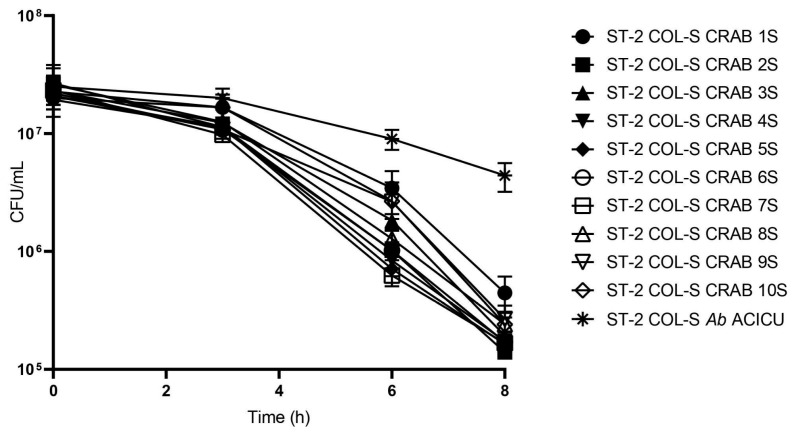
Persister assay CFU/mL regrowing cells in free-COL MHA medium. **Legend:** the data points represent the mean values of three independent biological replicates. Error bars indicate the standard deviation (SD) for each time point.

In clinical ST2 COL-S CRAB 1S–10S, a 2-log reduction (~10^7^ → 10^5^ CFU/mL) in viable cell counts (CFU/mL) was observed within 8 h of exposure to 5× MIC COL (Figure 2), while a reduction of only one log (~10^7^ → 10^6^ CFU/mL) was recovered in ST2 ACICU *Ab* within 8 h of observation (Table 1).

Statistical analysis (two-way ANOVA) demonstrated a statistically significant reduction in bacterial viability over 8 h in each strain when comparing T_0_ to the other time points (*p* < 0.0001). Statistically significant differences were not observed in the final persistence levels between the ten clinical isolates (*p* > 0.99, Tukey’s test). This uniformity suggests that the formation of a persistent subpopulation is a stable phenotypic trait of *A. baumannii* under colistin pressure. Conversely, a unique statistically significant difference in survival percentage was observed in *Ab* ACICU at T_8_, with a survival percentage of 17.6% and a one-log survival reduction (*p* < 0.05).

**Figure 3:**
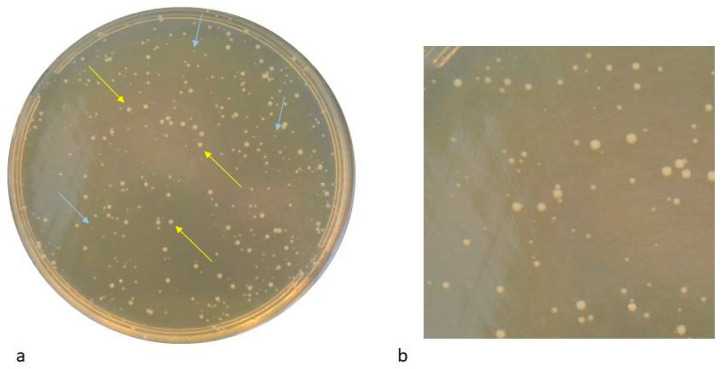
(**a**,**b**) Different colony morphologies in ST2 COL-S *Ab* ACICU regrowing cells after persister assay. **Legend:** (**a**) Yellow and blue arrows indicate the two different colony morphologies: big and small ones, respectively; (**b**) a focus on the plate to better distinguish big and small variants of the recovered persister subpopulation.

In general, two colony morphologies were observed in the regrown populations of all clinical ST2 COL-S CRAB and *Ab* ACICU Reference strain, as shown in Figure 3.

For both large and small colony phenotypes of the recovered ST2 COL-S CRAB 1S–10S and ST2 COL-S *Ab* ACICU isolates, COL MICs remained unchanged compared to parental strains.

### 2.3. Toxin/Antitoxin System Genomics

ST2 CRAB 1S and 2S were selected for T/A omic focus as hospital-dominant, globally disseminated, and well-characterized ST2 prototypes undergoing colistin persistence and in vivo colistin resistance under colistin pressure.

*TA Finder 2.0* prediction for type I-VIII T/A modules on chromosomal and plasmid-annotated ST2 COL-S CRAB 1S and 2S genomes revealed the presence of 11 T/A loci, as shown in Table 2.

All strains had the chromosomic type-II GNAT/DinJ, GNAT/HTH_31, Acetyltransf_4/HTH_24, AGP_acyltrn/panA, Doc/NpoA, HicA/B, and SRPBCC_CalC_Aha1-like/VapB T/As, and the single chromosomic type IV CptA/B T/A.

In addition, ST2 COL-S CRAB 1S and 2S had the plasmid type II BrnT/A module; on the contrary, the chromosomal type-II HigB/A was found only in the 2S CRAB.

Regarding the reference strain, the ST2 COL-S *Ab* ACICU was characterized by the additional presence of the chromosomal type II RelE/HicB family module.

### 2.4. Toxin/Antitoxin System Basal Transcriptomic Focus

A basal transcriptomic profiling of T/A systems revealed an expression landscape comprising eight shared chromosomal or plasmid T/A systems in both clinical ST2 COL-S CRAB 1S and 2S, having the same expression trend. Moreover, in each T/A system, the mRNA level of the toxin was consistently lower than the respective antitoxin. Notably, the absence of detectable toxin or antitoxin expression (RPKM = 0) in some modules could be due to levels of transcription below the detection threshold.

Concerning the expression of the shared T/As with the same trend, the highest expression level for both toxin and antitoxin was observed in the plasmid-encoded BrnT/A system (inferred both by plasmid assembly and annotation), which is involved in plasmid maintenance, stability, and persistence.

Among chromosomal modules, we found a novel dual combined (hybrid) II-type T/A–T/AasRNA regulatory model. In detail, an h-IIt (hybrid-type-II/toxin-asRNA) regulatory model was discovered for the GNAT/DinJ and AGP_acyltrn/PanA systems, in which toxin mRNA was associated with toxin small-antisense RNAs (asRNAs) and antitoxin mRNA. In these modules, asRNA GNAT (276–290 RPKM) and asRNA AGP (31–211 RPKM) transcripts exceed the corresponding mRNA toxins and antitoxins. A similar trend was observed for the HicA/B module, where only the HicA toxin asRNA (64–167 RPKM) was detected, while the antitoxin HicB remained transcriptionally silent (0 RPKM).

Conversely, GNAT/HTH_31 and CptA/B systems displayed a dual combined II type “h-IIa” (hybrid-type-II/antitoxin-asRNA) regulatory model characterized by prominent antitoxin asRNA transcription associated with toxin and antitoxin mRNAs. In GNAT/HTH_31, multiple antitoxin asRNAs were detected with values reaching 757 RPKM, whereas in the CptA/B system, the asRNA CptB (147–230 RPKM) significantly outweighed the mRNA of both CptA/B.

Finally, a classic T/A type-II with an exclusive antitoxin mRNA expression pattern was observed for the Doc/NpoA and AHA1/VapB systems. In these modules, toxin expression was completely absent (0 RPKM), while the corresponding antitoxins, NpoA and VapB, showed low but detectable transcription levels ranging from 1 to 40 RPKM in both 1S and 2S (Table 3).

Beyond the eight shared systems, the other 3 T/A modules—Acetyltransf_4/HTH_24, SRPBCC_CalC_Aha1-like/VapB, and COG4683/HTH_37—showed a strain-dependent expression trend.

## 3. Discussion

The ability of CRAB to withstand last-resort antimicrobials is a major clinical concern [19]. As previously described [4,5], the ten clinical ST2 COL-S CRAB rapidly evolved high-level COL resistance under in vivo therapy, with COL MICs increasing from 2 mg/L to 128 mg/L and stabilizing in the resulting ST2 COL-R CRAB isolates.

This transition to a “homogeneous-resistant” and “fully resistant” phenotype supports the notion that colistin monotherapy exerts strong selective pressure favoring the emergence of fixed, stable resistance traits in *Ab*. The predictable evolution of in vivo resistance underscores the importance of understanding subpopulations capable of surviving initial colistin exposure, including persister cells [11,20,21].

Addressing this critical issue, our study demonstrated that, despite being classified as susceptible to colistin, clinical COL-S CRAB strains (1S–10S) and the reference ST2 COL-S *Ab* ACICU retained the ability to survive normally high antimicrobial exposure by adapting their metabolism and entering into a dormant state.

The persistence assay revealed the presence of a surviving subpopulation able to persist under high-dose COL exposure, independent of their biofilm formation ability. Although COL exerts rapid bactericidal activity by destabilizing the outer membrane via interaction with lipopolysaccharides [22], the killing curves of all ST2 COL-S CRAB showed a progressive and incomplete survival decline over the 8 h observation. This profile, characterized by a rapid initial killing of the susceptible bulk population, followed by a plateau of persistent survivors, represented the typical biphasic curve signature of persisters [6,8,23].

All strains showed a time-dependent reduction in regrowth capacity, with survival rates decreasing from 85.0 to 40% at T_3_ to just 2.2–0.5% at T_8_. These findings suggested that even within highly drug-susceptible COL-S backgrounds, a small but resilient persister subpopulation exists and may serve as a reservoir for subsequent in vivo resistance development [24,25].

The same trend was observed for the reference strain ST2 COL-S *Ab* ACICU, which is also known to acquire in vivo colistin resistance, showing the highest persistence rates, with 80% survival at T_3_ and nearly 18% of the persister subpopulation still detectable at T_8_. This robust persistence phenotype may reflect strain-specific physiological or genetic attributes that promote tolerance under antimicrobial stress, such as alterations in membrane architecture, enhanced repair mechanisms, or metabolic reprogramming [26,27]. The temporal dynamics of COL persistence observed in the ST2 COL-S *Ab* ACICU strain—i.e., its higher persistence rate at extended time points (17.6% at T_8_)—seem to indicate a prolonged survival capacity. Given that the early bactericidal kinetics of colistin are comparable across the tested strains, this sustained viability highlights the profound influence of temporal phenomena on measured persistence. A compelling genomic rationale for this prolonged survival time and higher persister percentage than the ten clinical CRABs could be linked to the absence of the plasmidic BrnT/A system (RelE superfamily) in the ST2 *Ab* ACICU, unlike the other CRABs possessing this module and exhibiting a lower persister percentage over time and a lower survival time. Thus, the continuous maintenance and regulated expression of such plasmidic toxin–antitoxin addiction modules inherently impose a substantial metabolic burden, requiring the host to continuously allocate energetic and biosynthetic resources. Consequently, the lack of the *brnT/A* operon likely would mitigate these severe physiological constraints. By relieving the bacterial cell of the allostatic load and potential host–plasmid conflict associated with T/A maintenance, global cellular fitness is significantly enhanced. This energetic optimization provides a strong mechanistic framework to explain how the ST2 *Ab* ACICU persister subpopulation sustains its viability over prolonged periods, ultimately resulting in increased survival under sustained antimicrobial pressure [28].

The regrown cells derived from persisters, across all isolates and regardless of their colony morphology, maintained the same COL MICs as their parental strains, confirming that persistence, rather than hetero-resistance or rapid mutational resistance, accounts for survival during short-term high-dose exposure. This distinction is essential because persisters provide a non-genetic survival mechanism that may precede or facilitate the emergence of transiently resistant variants during therapy, acting as a bridge for the evolutionary acquisition of resistance [6].

To decipher the omic molecular drivers putatively involved in dormancy, we investigated the genomic and the basal transcriptomic landscape of different T/As, known to be involved in the induction and maintenance of persistence, in two clinical ST2 COL-S CRAB 1S and 2S [16].

At the genomic level, a pool of 10 type-II and 1 type-IV T/As was recognized, impacting several cellular functions such as protein synthesis, mRNA stability, plasmid maintenance, and host–pathogen interaction. Biologically, the diverse arsenal of T/A modules detected in our strains pointed to a multi-targeted strategy that could induce dormancy, acting as potent metabolic brakes, targeting distinct steps to ensure a complete shutdown of cells [16].

In particular, the chromosomal T/A network showed a marked prevalence of the GNAT superfamily, represented by the GNAT/DinJ, Acetyltransf_4/HTH_24, AGP_acyltrn/PanA, and GNAT/HTH_31 T/A modules. Despite their genetic diversity, these toxins share a conserved enzymatic activity as GCN5-related N-acetyltransferases and target the translational machinery via selectively acetylating aminoacyl-tRNAs, thereby preventing the loading of amino acids on the ribosome and stalling protein elongation and therefore translation. This could lead to a reversible block of growth, determining “enhanced survival”, and a modulated cell growth in response to stress, characterizing a persistent state [12,29,30]. In our CRABs, the prevalence of GNAT-related modules, such as GNAT/DinJ and GNAT/HTH_31, highlights an evolutionary preference for translational control via tRNA modification. Despite their genetic diversity, these modules share a unified functional output: the acetylation of aminoacyl-tRNAs. This specific enzymatic intervention prevents amino acids from entering the ribosome, putatively enabling the cell to swiftly halt translation and adjust protein synthesis in response to chromosomal T/A activation.

This translational blockade is also supported by the biological role of the Doc/NpoA system, inhibiting the elongation factor EF-Tu [31], and via the RNase activity of AHA1/VapB and HicA/B toxins [32,33,34]. These enzymes cleave specific mRNA or tRNA sequences, effectively removing the pool of translatable transcripts.

Moreover, the type-IV CptA/B module targets the bacterial cytoskeleton, inhibiting the polymerization of FtsZ and MreB proteins and physically blocking cell division and elongation [35].

In addition, a distinct and critical biological function was described for the plasmidic BrnT/A system (RelE superfamily). The BrnT toxin acts as a ribosome-dependent RNase, cleaving mRNAs at the ribosomal A-site. Its location on resistance plasmids imparts a dual function: besides its role in translation and, consequently, in dormancy, it serves as a plasmid-stabilization mechanism via post-segregational killing [36,37]. Selective elimination of daughter cells that fail to inherit the plasmid enforces the vertical transmission of resistance genes, preventing loss of the acquired resistome within the expanding population [38].

To evaluate the basal activity of T/A in XDR *Ab*-evolving COL persistence and in vivo full resistance under high COL exposure, we analyzed the basal transcriptomic landscape, under drug-free conditions, in two clinical, hospital-dominant, globally disseminated, and well-characterized ST2 COL-S CRAB prototypes.

Our analysis revealed marked basal transcriptional activity of the eight shared T/A networks, with consistently low toxin mRNA levels in the drug-free condition, as expected. Six chromosomal shared T/A systems and the plasmidic T/A system (BrnT/A) interfere with translation. In addition, the latter is involved in plasmid stabilization and transmissibility, whilst the chromosomal type-IV CptA/B also modulates transmissibility with cell division, as previously published [35]. It is particularly impactful that translation and the low-toxicity expression are the main shared targets across all functional T/A systems. This could suggest that the type-II GNAT superfamily translation-related T/A systems, flanked by the cell-division type-IV CptA/B, function as the primary molecular switches and balance regulators of the transition between vital and dormant cells. By modulating translation rather than inducing outright cell death, these systems allow cells to transiently slow growth without compromising viability. As already active under drug-free conditions, they can promptly modulate transcription rates, leading to dormancy in genomic backgrounds that can become fully colistin-resistant. This could lead to a reservoir of metabolically dormant cells, facilitating persistence and, under selective pressure in vivo, promoting the development of antibiotic resistance. Such coordination ensures population-level adaptability, balancing survival, plasmid maintenance, and stress responsiveness while minimizing fitness costs.

Similarly, the highest transcriptional expression rate was observed in the type-II plasmid-encoded BrnT/A module, showing robust co-expression of both toxin and antitoxin with a classical type-II pattern consistent with its canonical role in plasmid addiction and post-segregational killing. This suggested that the plasmid maintained could be a priority for the cell, ensuring stable inheritance of the resistant plasmid during both growth and persistence.

This strategy could ensure population-level adaptability, balancing survival, plasmid maintenance, and stress responsiveness while minimizing fitness costs.

At the molecular level, by transcriptomic-based inferences, we first described novel fine-tuned T/A transcript balance regulatory models. Unlike the classical model of protein-protein inhibition of T/A type-II/IV, our data highlight an alternative dual combined T/A mRNA-asRNA balance-regulatory model, in which asRNAs emerged as toxin and antitoxin transcript ratio balance regulators, regulating the amounts and hence the prevalence of the specific T or A transcripts, despite previously being classified as type-II and -IV models. Balancing the T/A mRNA-asRNA ratio, they could speculatively switch on/off T/A system activity, leading to dormancy or maintaining a metabolically active cell state, respectively.

Systems such as GNAT/DinJ, AGP_acyltrn/PanA, and HicA/B displayed a predominant asRNA toxin modulation. In these loci, high levels of antisense transcripts targeting the toxin genes sharply contrasted with the low or undetectable levels of their cognate sense antitoxin mRNAs, indicating a toxin-centered regulation governed at the RNA level by their cis asRNAs. This is particularly relevant for modules such as the AGP_acyltrn/PanA, where the toxin is a 1-acyl-sn-glycerol-3-phosphate acyltransferase that also acts as a metabolic enzyme in membrane glycerophospholipid synthesis [39]. Similarly, the GNAT toxin is an unusual histone acetyltransferase involved epigenetically as a virulence factor, mediating complex host–pathogen interactions. The HicA toxin, a potent translation inhibitor through its mRNA-cleaving RNase activity, followed this same pattern: its antisense-dominated profile suggests a tight post-transcriptional control that prevents accidental growth arrest while maintaining the toxin in a poised state.

The prevalence of toxin-targeted asRNAs across these diverse modules suggested that in drug-free growth, these ST2 COL-S CRAB strains can predominantly rely on RNA-level interference rather than traditional protein-based antitoxins to regulate toxin activity. This configuration functionally couples metabolic maintenance and virulence with a built-in potential for rapid switching to persistence once a stress signal, such as colistin exposure, is encountered.

A distinct but still asRNA-based regulatory strategy was observed for the GNAT/HTH_31 and CptA/B modules, in which antisense transcription was primarily directed against the antitoxin components. In the GNAT/HTH_31 system, the transcriptomic profile was dominated by three distinct asRNAs targeting the HTH_31 antitoxin, reaching high levels and suggesting multilayered repression of the antitoxin that may fine-tune GNAT toxin availability. Similarly, in the CptA/B module, an abundant antisense transcript targeting CptB indicated that antitoxin expression is tightly controlled at the RNA level, potentially lowering the threshold for toxin activation under stress and contributing to a flexible, rapidly inducible dormancy response.

Finally, the Doc/NpoA and AHA1/VapB modules presented an “antitoxin-independent” signature, with complete toxin silencing alongside low but detectable antitoxin expression. This basal antitoxin accumulation can represent a preventive shield strategy: NpoA actively neutralizes the kinase activity of Doc (which phosphorylates EF-Tu to block translation), while VapB sequesters the RNase AHA1/VapC to preserve mRNA integrity during active growth.

This diverse expression landscape revealed an active basal transcription of multiple T/As, ranging from balanced co-expression (BrnT/A) to asRNA-dominated (GNAT/DinJ, HicA/B, GNAT/HTH_31, CptA/B) and antitoxin-independent (Doc/NpoA, AHA1/VapB) patterns, leading to a low toxin RNA expression.

Although our study provides a detailed molecular and transcriptomic characterization of ST2 COL-S CRAB persistence, it is limited by its focus on in vitro assays under drug-free conditions and on a small subset of representative strains. Functional validation of the novel T/A-asRNA regulatory networks remains indirect, and the in vivo relevance of these mechanisms requires further confirmation. Therefore, extending these analyses to conditions with antimicrobial exposure represents a critical next step to directly assess how T/A modules respond under therapeutic pressure and to better predict the emergence of stable colistin resistance. Furthermore, while these models are currently based on robust transcriptomic patterns and genomic co-localization, they should be considered as functional frameworks rather than experimentally validated mechanisms. Given the unprecedented nature of these TA–asRNA interactions, further biochemical studies, such as in vitro binding assays or targeted asRNA deletions, will be essential to definitively confirm the physical and functional crosstalk between these components.

In conclusion, integrating the biological and molecular aspects of our results, these findings establish the molecular basis for the observed persistence in colistin-susceptible (COL-S) ST2 CRAB, linking a diverse, transcriptionally active htype-II GNAT superfamily Toxin/Antitoxin (T/A) repertoire—including novel T/A-asRNA models—to the phenotypic persistence that precedes in vivo evolution toward stable, high-level colistin resistance. Basal expression positions these strains in a transcriptionally primed state, with tightly controlled toxin levels under drug-free conditions, effectively creating a dormancy-ready molecular landscape. Co-localization of the BrnT/A module on an XDR plasmid further ensures plasmid retention during severe metabolic shutdown, generating a translationally arrested survival reservoir. This immediate survival window provides the residual population with the time necessary to acquire and fix genetic adaptations, bridging transient persistence to genetic adaptation and explaining the rapid in vivo transition from colistin susceptibility to pandrug resistance.

## 4. Materials and Methods

### 4.1. Bacterial Strains and Antimicrobial Susceptibility

Ten clinical ST2 XDR COL-S CRAB 1S–10S and an ST2 COL-S *Ab* ACICU reference strain were included in the study. XDR CRABs were isolated from patients in Catania hospitals. The ten unrelated Italian COL-S clinical CRABs (1-S/10-S) were recovered from bronchial aspirates of patients hospitalized in Intensive Care Units (ICU) at different Sicilian hospitals while receiving colistin.

Alongside the previously characterized isolates 1S and 2S [5], eight additional ST2 COL-S CRAB clinical strains (3S–10S) were included to investigate the persistence mechanism.

As previously published [5], ST2 COL-susceptible CRAB strains 1S and 2S acquired COL resistance during colistin therapy, evolving into ST2 COL-resistant CRAB strains 1R and 2R. All clinical isolates in this study, as well as the ST2 COL-S *Ab* ACICU reference strain, evolved in vivo during therapy in COL-R counterparts, achieving a stable, high COL MIC of 128 mg/L, delineating the ‘homogeneous-resistant’ and “Full Resistance” phenotypes [5].

Strains were grown in MacConkey (Oxoid, Basingstoke, UK) agar plates at 37 °C for 18 h and identified by Matrix-Assisted Laser Desorption Ionization–Time of Flight (MALDI-TOF) as previously published [5].

Antimicrobial resistance and susceptibility to COL, imipenem (IMP), meropenem (MEM), ampicillin/sulbactam (SAM), ciprofloxacin (CIP), gentamicin (GEN), amikacin (AK), tigecycline (TGC), and trimethoprim/sulfamethoxazole (SXT) were tested using the Minimum Inhibitory Concentration (MIC) broth microdilution assay according to the EUCAST 2025 guidelines [40]. Results were assessed visually by identifying the lowest antibiotic concentration that completely inhibited visible bacterial growth.

### 4.2. Biofilm Production

Biofilm production assays were performed in Trypton Soy Broth (TSB) (Oxoid, Basingstoke, UK) supplemented with 5% glucose in technical triplicates and five biological replicates as previously published [41]. The assays were considered negative for OD_655_ < 0.04 according to the blank value: 0.043 ± 0.002611, expressed as mean ± SD.

### 4.3. Persister Selection Assay

A persister selection assay was carried out in biological triplicates to ensure the reproducibility of the results, as previously described with some modifications [42]. Briefly, COL-S strains have been grown in Luria–Bertani broth (LB) (Difco, BD Biosciences, Franklin Lakes, NJ, USA) since the exponential phase (OD_600_ = 0.5). Thus, each sample was exposed to colistin as follows: ~10^7^ CFU/mL of each standardized culture was exposed to a colistin concentration 5-fold MIC COL in LB at 37 °C with shaking for 8 h. At three (T_3_), six (T_6_), and eight hours (T_8_) of incubation, OD_600_ was measured, cultures were washed once in sterile H_2_O, and spread on Mueller-Hinton agar (Oxoid, Basingstoke, UK) plates. Then, plates were incubated at 37 °C for 24 h, and CFU/mL were evaluated, and COL-MICs were performed to ensure the MIC value had not changed.

### 4.4. Statistical Analysis

Data are presented as the mean SD of three independent experiments. Bacterial viability was expressed as log_10_ CFU/mL for all statistical comparisons. Statistical significance was assessed by two-way ANOVA followed by Tukey’s multiple comparisons test using GraphPad Prism 9.0. A *p*-value < 0.05 was considered statistically significant.

### 4.5. Whole Genome Sequencing (WGS)

Whole Genome Sequencing (WGS) was performed for the clinical ST2 XDR COL-S CRAB 1S and 2S as previously published [4,5].

Briefly, genomic DNA was extracted using the PureLink Genomic DNA Mini Kit (Invitrogen, Waltham, MA, USA) according to the manufacturer’s protocol. DNA quality was assessed using Qubit, and its concentration was determined by Picogreen (Life Technologies, Carlsbad, CA, USA). Whole Genome Sequencing (WGS) was achieved with the Illumina Mi-seq 300P sequencing system using paired-end (PE) read libraries prepared by the Nextera XT DNA Library Preparation Kit (Illumina, San Diego, CA, USA) following the manufacturer’s protocol, and the quality was quantified as previously published [4,5]. The indexed libraries were evaluated as previously published [4,5] pooled at a final concentration of 2 nM and used for Illumina MiSeq sequencing with a PE 300 (2 × 300 bp). Raw reads were processed with QUAST (v.4.6.3) for quality evaluation, and the Cutadapter tool (v.1.16) implemented in Python (v.3.5.2) was employed to remove residual PCR primers, low-quality (Q_score < 30), and short reads (<150 bp). The trimmed reads were used for downstream analysis.

### 4.6. Genome Assembly

De novo genome assembly was performed with the SPAdes software (v.3.12.0), generating contig files for each sample. Post-assembly metrics were evaluated by QUAST (v.4.6.3) as previously published [4,5].

### 4.7. Genome Annotation

Genome annotations were performed by CDSeasy (v1.0). A tool that quickly performs protein-coding gene identification with Prodigal V2.60 and the initial functional annotation using Blastp (v2.17.0) against the bacterial database Representative Proteomes RP55 [43].

### 4.8. T/A Finder

Toxin/antitoxin systems were predicted by T/A *finder 2.0*, a web-based tool to identify type I-VIII toxin–antitoxin loci in bacterial genomes [44]. Confirmation of T/A modules was obtained manually by tBLASTn (v2.17.0+).

### 4.9. RNA Seq

#### 4.9.1. RNA-Seq Sample

Among the ten clinical CRAB isolates, strains 1S and 2S were selected for transcriptomic profiling as hospital-dominant, globally disseminated, and genomically well-characterized prototypes evolving in vivo colistin persistence as well as resistance under therapeutic pressure.

#### 4.9.2. RNA Extraction and Sequencing

RNA extraction and sequencing were performed for the clinical ST2 XDR COL-S CRAB 1S and 2S as previously published [4]. Briefly, RNA-seq was performed on two biological replicates per strain using the Illumina MiSeq Standard pipeline. Two distinct strand-specific RNA-seq libraries were sequenced for each strain: a 50 bp single-end (short insert) library and a 150 bp paired-end (TruSeq) library.

### 4.10. Tru-Seq and Short Insert Library Analysis

TS and SI RNA-seq reads were aligned on the *Ab* ACICU (CP000863.1) Reference Genome. Assembly and quantification were performed using Rockhopper v.2.03, a tool specifically designed to investigate bacterial gene structures and transcriptomes and validated for identifying novel sRNAs [4].

A focus of basal profiling transcriptomics in the drug-free condition of two biological replicates at the mid-exponential growth phase was performed to obtain a comprehensive basal transcriptional profile of the entire T/A system pool. This approach led to the use of RNA-seq to map and quantify transcripts, including antisense RNAs and small RNAs, in a basic profiling manner, without comparing different conditions [45,46].

Expression was expressed as RPKM values, which were calculated to normalize for gene length and library size, ensuring comparability between libraries. All samples passed quality control, and normalized read counts were used for downstream analyses.

We classified and indicated “h-IIt”, a T/A regulatory module of type II with toxin/antitoxin mRNAs and toxin asRNA, as well as “h-IIa” with the simultaneous presence of toxin and antitoxin mRNAs and antitoxin asRNA.

## Figures and Tables

**Table 1:** ST2 COL-S CRAB 1S–10S and ST2 COL-S *Ab* ACICU persister survival under high COL exposure.

ST2 COL-S CRAB/*Ab*	T_0_ (CFU/mL)	T_3_ (CFU/mL)	T_6_ (CFU/mL)	T_8_ (CFU/mL)	T_3_% Survival	T_6_% Survival	T_8_% Survival
1S	2.0 ± 0.1 × 10^7^	1.7 ± 0.4 × 10^7^	3.4 ± 1.4 × 10^6^	4.4 ± 1.1 ×10^5^	85.0%	17.0%	2.2%
2S	2.7 ± 0.9 × 10^7^	1.1 ± 0.2 × 10^7^	1.0 ± 0.5 × 10^5^	1.4 ± 0.2 × 10^5^	40.7%	3.7%	0.5%
3S	2.2 ± 0.3 × 10^7^	1.3 ± 0.2 × 10^7^	2.1 ± 0.3 × 10^6^	1.7 ± 0.2 × 10^5^	59.1%	9.5%	0.8%
4S	2.0 ± 0.2 × 10^7^	1.0 ± 0.1 × 10^7^	8.4 ± 0.9 × 10^5^	1.6 ± 0.3 × 10^5^	50.0%	4.2%	0.8%
5S	2.3 ± 0.6 × 10^7^	1.1 ± 0.2 × 10^7^	7.4 ± 0.8 × 10^5^	1.6 ± 0.4 × 10^5^	47.8%	3.2%	0.7%
6S	2.1 ± 0.2 ×10^7^	1.1 ± 0.1 × 10^7^	7.1 ± 0.8 × 10^5^	1.6 ± 0.2 × 10^5^	52.4%	3.4%	0.8%
7S	2.1 ± 0.2 × 10^7^	1.1 ± 0.1 × 10^7^	1.0 ± 0.1 × 10^6^	2.0 ± 0.2 × 10^5^	52.4%	4.8%	1.0%
8S	1.9 ± 0.2 × 10^7^	9.8 ± 0.9 × 10^6^	6.2 ± 0.6 × 10^5^	1.4 ± 0.2 × 10^5^	51.6%	3.3%	0.7%
9S	2.1 ± 0.2 × 10^7^	1.3 ± 0.1 × 10^7^	1.3 ± 0.1 × 10^6^	2.4 ± 0.1 × 10^5^	61.9%	6.2%	1.1%
10S	2.2 ± 0.5 × 10^7^	1.4 ± 0.2 × 10^7^	2.6 ± 0.6 × 10^6^	2.3 ± 0.6 × 10^5^	63.6%	11.8%	1.0%
*Ab* ACICU	2.5 ± 0.5 × 10^7^	2.0 ± 0.3 × 10^7^	8.2 ± 2.2 × 10^6^	4.4 ± 1.1 × 10^6^	80.0%	32.8%	17.6%

**Legend:** Values are expressed as mean ± standard deviation (SD) of three independent biological replicates. Survival percentages were calculated relative to the initial inoculum at T_0_.

**Table 2:** Predicted T/A systems in clinical ST2 COL-S CRAB 1S-2S and ST2 COL-S *Ab* ACICU.

T/A-Type	Localization	T/A Family	Protein Domain	ST2 COL-S CRAB 1	ST2 COL-S CRAB 2	ST2 COL-S *Ab* ACICU
II	Chromosome	GNAT/relB	GNAT/DinJ	+	+	+
II	Chromosome	-	GNAT/HTH_31	+	+	+
II	Chromosome	-	Acetyltransf_4/HTH_24	+	+	+
II	Chromosome	-/panA	AGP_acyltrn/panA	+	+	+
II	Chromosome	doc/NpoA	Doc/NpoA	+	+	+
II	Chromosome	hicA/hicB	HicA/HicB	+	+	+
IV	Chromosome	cptA/cptB	CptA/CptB	+	+	+
II	Chromosome	-/vapB	SRPBCC_CalC_Aha1-like/vapB	+	+	+
II	Plasmid	brnT/brnA	BrnT/BrnA	+	+	-
II	Chromosome	higB/higA	COG4683/HTH_37	-	+	-
II	Chromosome	relE/hicB	-/HicB	-	-	+

**Legend:** +: presence of the T/As; -: absence of the T/As.

**Table 3:** T/A shared expression in ST2 COL-S 1S and 2S CRAB.

Expression	Type	T/A Module	Ref.Gen. ACICU	Product	RPKM 1S	RPKM 2S
Toxin/Antitoxin	II	BrnT/A	-	BrnT	1329	2523
			-	BrnA	6575	5907
Toxin/Antitoxin and toxin asRNA	h-IIt	GNAT/DinJ	ACICU_02316	GNAT	161	115
			ACICU_02316	Antisense GNAT	276	290
			ACICU_02315	DinJ	17	21
		AGP_acyltrn/PanA	ACICU_00518	AGP_acyltrn	23	12
			ACICU_00518	Antisense AGP	211	31
			ACICU_00519	PanA	5	6
		HicA/HicB	ACICU_02726	Antisense HicA	64	167
			ACICU_02725	HicB	0	0
Toxin/Antitoxin and antitoxin asRNA	h-IIa	GNAT/HTH_31	ACICU_01559	GNAT	6	5
			ACICU_01558	HTH_31	8	16
			ACICU_01558	Antisense HTH_31	253	120
			ACICU_01558	Antisense HTH_31	144	198
			ACICU_01558	Antisense HTH_31	757	317
		CptA/B	ACICU_03248	CptA	9	6
			ACICU_03249	CptB	16	15
			ACICU_03249	Antisense CptB	147	230
Antitoxin only	II	Doc/NpoA	ACICU_00915	Doc	0	0
			ACICU_00916	NpoA	1	15
		AHA1/VapB	ACICU_03070	AHA1	0	0
			ACICU_03071	VapB	40	31

**Legend:** - absence of Ref.Gen. ACICU locus tag.

## Data Availability

The genomic sequence reads were deposited in the NCBI Genome database in the Sequence Read Archive (SRA) under study accession n° SRP133297. The transcriptomic datasets analyzed for this study are available in the NCBI GEO Database (GSE109951).

## References

[1] Sharma R., Lakhanpal D. (2025). *Acinetobacter baumannii*: A Comprehensive Review of Global Epidemiology, Clinical Implications, Host Interactions, Mechanisms of Antimicrobial Resistance and Mitigation Strategies. Microb. Pathog..

[2] WHO Publishes List of Bacteria for Which New Antibiotics Are Urgently Needed. https://www.who.int/news/item/27-02-2017-who-publishes-list-of-bacteria-for-which-new-antibiotics-are-urgently-needed/.

[3] Mohapatra S.S., Dwibedy S.K., Padhy I. (2021). Polymyxins, the Last-Resort Antibiotics: Mode of Action, Resistance Emergence, and Potential Solutions. J. Biosci..

[4] Cafiso V., Stracquadanio S., Lo Verde F., Dovere V., Zega A., Pigola G., Aranda J., Stefani S. (2019). COLR *Acinetobacter baumannii* sRNA Signatures: Computational Comparative Identification and Biological Targets. Front. Microbiol..

[5] Cafiso V., Stracquadanio S., Dovere V., Lo Verde F., Zega A., Pigola G., Barnini S., Ghelardi E., Stefani S. (2021). Colistin Resistance Onset Strategies and Genomic Mosaicism in Clinical *Acinetobacter baumannii* Lineages. Pathogens.

[6] Brauner A., Fridman O., Gefen O., Balaban N.Q. (2016). Distinguishing between Resistance, Tolerance and Persistence to Antibiotic Treatment. Nat. Rev. Microbiol..

[7] Levin-Reisman I., Ronin I., Gefen O., Braniss I., Shoresh N., Balaban N.Q. (2017). Antibiotic Tolerance Facilitates the Evolution of Resistance. Science.

[8] Balaban N.Q., Helaine S., Lewis K., Ackermann M., Aldridge B., Andersson D.I., Brynildsen M.P., Bumann D., Camilli A., Collins J.J. (2019). Definitions and Guidelines for Research on Antibiotic Persistence. Nat. Rev. Microbiol..

[9] Goormaghtigh F., Van Melderen L. (2019). Single-Cell Imaging and Characterization of *Escherichia coli* Persister Cells to Ofloxacin in Exponential Cultures. Sci. Adv..

[10] La Rosa R., Johansen H.K., Molin S. (2022). Persistent Bacterial Infections, Antibiotic Treatment Failure, and Microbial Adaptive Evolution. Antibiotics.

[11] Niu H., Gu J., Zhang Y. (2024). Bacterial Persisters: Molecular Mechanisms and Therapeutic Development. Signal Transduct. Target. Ther..

[12] Jurėnas D., Fraikin N., Goormaghtigh F., Van Melderen L. (2022). Biology and Evolution of Bacterial Toxin–Antitoxin Systems. Nat. Rev. Microbiol..

[13] Maisonneuve E., Gerdes K. (2014). Molecular Mechanisms Underlying Bacterial Persisters. Cell.

[14] Gerdes K., Christensen S.K., Løbner-Olesen A. (2005). Prokaryotic Toxin–Antitoxin Stress Response Loci. Nat. Rev. Microbiol..

[15] Koga M., Otsuka Y., Lemire S., Yonesaki T. (2011). *Escherichia coli rnlA* and *rnlB* Compose a Novel Toxin–Antitoxin System. Genetics.

[16] Gerdes K., Maisonneuve E. (2012). Bacterial Persistence and Toxin-Antitoxin Loci. Annu. Rev. Microbiol..

[17] Zhang S.-P., Wang Q., Quan S.-W., Yu X.-Q., Wang Y., Guo D.-D., Peng L., Feng H.-Y., He Y.-X. (2020). Type II Toxin–Antitoxin System in Bacteria: Activation, Function, and Mode of Action. Biophys. Rep..

[18] Magiorakos A.-P., Srinivasan A., Carey R.B., Carmeli Y., Falagas M.E., Giske C.G., Harbarth S., Hindler J.F., Kahlmeter G., Olsson-Liljequist B. (2012). Multidrug-Resistant, Extensively Drug-Resistant and Pandrug-Resistant Bacteria: An International Expert Proposal for Interim Standard Definitions for Acquired Resistance. Clin. Microbiol. Infect..

[19] Thacharodi A., Vithlani A., Hassan S., Alqahtani A., Pugazhendhi A. (2024). Carbapenem-Resistant *Acinetobacter baumannii* Raises Global Alarm for New Antibiotic Regimens. iScience.

[20] Kunnath A.P., Suodha Suoodh M., Chellappan D.K., Chellian J., Palaniveloo K. (2024). Bacterial Persister Cells and Development of Antibiotic Resistance in Chronic Infections: An Update. Br. J. Biomed. Sci..

[21] Hashemi M.J., Dhaouadi Khattab Y., Ren D. (2025). Mini Review: Persister Cell Control Strategies. Front. Pharmacol..

[22] Andrade F.F., Silva D., Rodrigues A., Pina-Vaz C. (2020). Colistin Update on Its Mechanism of Action and Resistance, Present and Future Challenges. Microorganisms.

[23] Alexandersen N.R., Nielsen K.L., Häussler S., Bjarnsholt T., Schønning K. (2025). Antibiotic Tolerance and Persistence in Clinical Isolates of *Escherichia coli* Evaluated by High-Resolution Time-Kill Assays. Microbiol. Spectr..

[24] Liu X., Tang R., Li H., Wang L., Wan C. (2022). The Physiological and Ecological Properties of Bacterial Persisters Discovered from Municipal Sewage Sludge and the Potential Risk. Environ. Res..

[25] Windels E.M., Michiels J.E., Fauvart M., Wenseleers T., Van Den Bergh B., Michiels J. (2019). Bacterial Persistence Promotes the Evolution of Antibiotic Resistance by Increasing Survival and Mutation Rates. ISME J..

[26] Iacono M., Villa L., Fortini D., Bordoni R., Imperi F., Bonnal R.J.P., Sicheritz-Ponten T., De Bellis G., Visca P., Cassone A. (2008). Whole-Genome Pyrosequencing of an Epidemic Multidrug-Resistant *Acinetobacter baumannii* Strain Belonging to the European Clone II Group. Antimicrob. Agents Chemother..

[27] Hamidian M., Wick R.R., Hartstein R.M., Judd L.M., Holt K.E., Hall R.M. (2019). Insights from the Revised Complete Genome Sequences of *Acinetobacter baumannii* Strains AB307-0294 and ACICU Belonging to Global Clones 1 and 2. Microb. Genom..

[28] Yang Q.E., Walsh T.R. (2017). Toxin–Antitoxin Systems and Their Role in Disseminating and Maintaining Antimicrobial Resistance. FEMS Microbiol. Rev..

[29] Cheverton A.M., Gollan B., Przydacz M., Wong C.T., Mylona A., Hare S.A., Helaine S. (2016). A Salmonella Toxin Promotes Persister Formation through Acetylation of tRNA. Mol. Cell.

[30] Vetting M.W., de Carvalho L.P.S., Yu M., Hegde S.S., Magnet S., Roderick S.L., Blanchard J.S. (2005). Structure and Functions of the GNAT Superfamily of Acetyltransferases. Arch. Biochem. Biophys..

[31] Cruz J.W., Rothenbacher F.P., Maehigashi T., Lane W.S., Dunham C.M., Woychik N.A. (2014). Doc Toxin Is a Kinase That Inactivates Elongation Factor Tu. J. Biol. Chem..

[32] Hou B., Wang C.-Y., Li S.-W., Zhou L.-J., Che Y.-L., Chen Q.-Y. (2023). Effects of Toxin-Antitoxin System HicAB on Biofilm Formation by Extraintestinal Pathogenic *E. coli*. Curr. Microbiol..

[33] Encina-Robles J., Pérez-Villalobos V., Bustamante P. (2024). The HicAB System: Characteristics and Biological Roles of an Underappreciated Toxin-Antitoxin System. Int. J. Mol. Sci..

[34] Tripathi V., Darnauer S., Hartwig N.R., Obermann W.M.J. (2014). Aha1 Can Act as an Autonomous Chaperone to Prevent Aggregation of Stressed Proteins. J. Biol. Chem..

[35] ElBanna S.A., Moneib N.A., Aziz R.K., Samir R. (2021). Genomics-Guided Identification of a Conserved CptBA-like Toxin-Antitoxin System in *Acinetobacter baumannii*. J. Adv. Res..

[36] Jensen R.B., Gerdes K. (1995). Programmed Cell Death in Bacteria: Proteic Plasmid Stabilization Systems. Mol. Microbiol..

[37] Hayes F., Van Melderen L. (2011). Toxins-Antitoxins: Diversity, Evolution and Function. Crit. Rev. Biochem. Mol. Biol..

[38] Heaton B.E., Herrou J., Blackwell A.E., Wysocki V.H., Crosson S. (2012). Molecular Structure and Function of the Novel BrnT/BrnA Toxin-Antitoxin System of Brucella Abortus. J. Biol. Chem..

[39] Parsons J.B., Rock C.O. (2013). Bacterial Lipids: Metabolism and Membrane Homeostasis. Progress. Lipid Res..

[40] EUCAST. https://www.eucast.org/news-detail/updated-clinical-breakpoints-dosages-qc-and-methods-documents/.

[41] Granata G., Stracquadanio S., Consoli G.M.L., Cafiso V., Stefani S., Geraci C. (2019). Synthesis of a Calix [4]Arene Derivative Exposing Multiple Units of Fucose and Preliminary Investigation as a Potential Broad-Spectrum Antibiofilm Agent. Carbohydr. Res..

[42] Chung E.S., Ko K.S. (2019). Eradication of Persister Cells of *Acinetobacter baumannii* through Combination of Colistin and Amikacin Antibiotics. J. Antimicrob. Chemother..

[43] Li J., Tai C., Deng Z., Zhong W., He Y., Ou H.-Y. (2017). VRprofile: Gene-Cluster-Detection-Based Profiling of Virulence and Antibiotic Resistance Traits Encoded within Genome Sequences of Pathogenic Bacteria. Brief. Bioinform..

[44] Guan J., Chen Y., Goh Y.-X., Wang M., Tai C., Deng Z., Song J., Ou H.-Y. (2024). TADB 3.0: An Updated Database of Bacterial Toxin–Antitoxin Loci and Associated Mobile Genetic Elements. Nucleic Acids Res..

[45] Sharma C.M., Hoffmann S., Darfeuille F., Reignier J., Findeiß S., Sittka A., Chabas S., Reiche K., Hackermüller J., Reinhardt R. (2010). The Primary Transcriptome of the Major Human Pathogen *Helicobacter pylori*. Nature.

[46] Wurtzel O., Yoder-Himes D.R., Han K., Dandekar A.A., Edelheit S., Greenberg E.P., Sorek R., Lory S. (2012). The Single-Nucleotide Resolution Transcriptome of *Pseudomonas aeruginosa* Grown in Body Temperature. PLoS Pathog..

